# Effects of an Avatar Control on VR Embodiment

**DOI:** 10.3390/bioengineering12010032

**Published:** 2025-01-03

**Authors:** DoHyung Kim, Halim Yeo, Kyoungju Park

**Affiliations:** Department of Computer Science and Engineering, Chung-Ang University, Seoul 06974, Republic of Korea; skemailid@naver.com (D.K.); gkfla9411@naver.com (H.Y.)

**Keywords:** virtual reality, emotional responses, extended reality, head-mounted displays, character generation

## Abstract

The motion control of the virtual avatar enhances a sense of embodiment in a virtual reality (VR). Yet, the detailed relationship between motion control, assigned tasks, and the sense of embodiment remains unclear. We aim to investigate the relationships between degrees of control on a full-body avatar and three elements of the sense of embodiment: the sense of self-location, agency, and ownership in standalone and interaction tasks. To do this, we conducted a user study with three conditions of control over a full-body avatar. The types of control are (1) Low—control of an upper-body avatar, (2) Mid—control of a full-body avatar from three sensors, and (3) High—control of a full-body avatar from six sensors. These three control methods, which were used to animate the avatars and imitate the users’ pose, differ in accuracy and stability. Participants embodied three kinds of control and performed a single-user task (obstacle avoidance) and a multi-user task (catch-ball). Our results indicate that the degree of control impacts participants’ embodiment. However, there was no significant difference between high- and mid-control in the multi-user task, which was a different result from the single-user task. This suggests for virtual bodies that the participants capacity to control and see are the same or different, which may affect embodiment. Our result also shows that the multi-user task enhanced the sense of embodiment compared to the single-user task in the low- and mid-control avatars. Yet, the multi-user task decreased the sense of agency of the high-control avatar. This suggests that a failure of the assigned task may affect the sense of agency, especially when it is close to success, yielding revulsion. We further elucidate the insights into the relationship between the degree of control, the assigned tasks, and the elements of a sense of embodiment.

## 1. Introduction

Embodiment is the perception that a user has a body within the virtual world. Kilteni et al. describe this sense of embodiment as the feeling of “being inside, having, and controlling a body” in virtual reality (VR) [1]. Disembodied users feel quite present when given a virtual body that correctly matches theirs; they quickly realize that there are different levels of presence [2]. VR applications need to design avatars so that users feel embodied.

The process of making avatars is tailored by several technical constraints in VR, including motion capture capabilities and 3D model reconstruction. Therefore, developers require a set of choices to build a functional avatar and to know how users accept these choices, as well as how these factors affect their perception of the resulting avatar. Researchers have studied factors of influence, appearance, and control toward embodiments. Precisely, they investigated how these factors influence the sense of embodiment as the combination of a sense of self-location, sense of agency, and sense of ownership [1]. Yet, little has been studied regarding the influences of advanced motion control technology on the three elements of the sense of embodiment.

We aim to investigate the effects of an upper-body control avatar (low), a full-body control from three sensors (mid), and a full-body control from six sensors (high) on three elements of the sense of embodiment. A low-control avatar generates the upper body using inverse kinematics (IKs) from the headset and two hand-held controllers. A mid-control avatar reconstructs an entire body using deep learning. A high-control avatar captures an entire body from six sensors by attaching wearable trackers to the lower body of the users. These three kinds of motion control methods differ in accuracy and robustness. We investigates the relationship between control and embodiment when users performed tasks.

To provide insights, we present two experiments exploring users’ preferences and perceptions of three levels of full-body control on embodiment. The first is an obstacle-avoidance experiment in which a user controls his virtual avatar to avoid approaching box-size obstacles. The second is a catch-ball experiment in which two users throw and catch a volleyball-sized ball. Our user study collected participants’ answers in terms of three levels of control in the two experiments, evaluated them statistically, and validated our hypotheses. Through our user study, we investigated how the degrees of control and inter-relations with the assigned tasks affect the sense of embodiment in VR.

Our experimental results show that improving the degrees of control enhances the sense of embodiment, but there are exceptions. The mid- and high-control avatars showed no significant differences in the multi-user task. This suggests that participants accept the plausible control of others’ virtual bodies in the multi-user task, yielding no significant differences between the mid- and high-control avatars. A comparison of single- and multi-player tasks provides insights into the inter-relations of the control and assigned tasks regarding the sense of location, agency, and ownership. The analysis shows that social interaction in multi-user tasks enhances the sense of ownership most of the time in low- and mid-control avatars. However, the multi-user task yielded a controversial effect on the sense of agency, specifically decreasing the sense of agency of the high-control avatar. This suggests that participants experience revulsion when encountering failure in interaction when it seems possible to succeed.

To the best of our knowledge, we are the first study to incorporate the mid-control avatars of neural network regression and study the effects on the sense of embodiment. Also, we are the first to study the inter-relations of the control and the interaction tasks in a multi-user experiment regarding the three components of the sense of embodiment.

## 2. Related Work

### 2.1. Sense of Embodiment

Kilteni et al. address the sense of embodiment in VR [1] as a criterion to measure. The sense of embodiment has three elements: self-location, sense of agency, and sense of ownership.

Sense of self-location: Self-location is when one feels that one knows where they are located. With self-location, they recognize where they are and expect the consequences of their movements to be applied to a virtual body representation, which is a collocation between the virtual body representation and the real body. However, this collocation breaks down when people have out-of-body experiences in which they perceive themselves outside of their physical body [3]. Also, self-location is influenced by the visuospatial perspective [4]. Furthermore, vestibular signals also play a crucial role when one determines self-location.

Sense of agency: A sense of agency is defined as one’s perception that one’s actions contribute to one’s virtual body representation. When interacting with our bodies, we accurately control our activity and are aware of our actions [5]. The sense of agency is closely related to action awareness and action plans. The sense of agency focuses on the action of the player. Tsakiris et al. [6] address that the sense of agency plays a crucial role in the sense of ownership, since the action plan leads directly to the self-awareness of body ownership. A sense of agency is measured by examining whether the participant perceives high control over the virtual representation.

Sense of ownership: The sense of ownership is defined as a sense that one’s own body perceives or feels the same as the virtual body representation. The sense of ownership is influenced by self-location and a sense of agency. In other words, the sense of ownership is affected by similar appearance, similar movement, and that it shares sensation [7]. The sense of ownership assumes participants ultimately consider virtual body representation as their extension of sensation, and the sense of ownership encompasses a sense of agency and self-location to some extent [8].

Researchers also have conducted experiments that compare different virtual body representations or different virtual body movement methods to discuss the embodiment aspect. Ferran Argelaguet conducted experiments to assess how embodiment is enhanced based on the similarity of virtual hands in VR to real hands by dividing them into abstract, iconic, and realistic categories [9]. Lougiakis, C researched how embodiment differs when virtual hands in VR are similar to real hands versus resembling VR controller devices [10]. M. Parger investigated how the implementation of virtual arm movement in VR, based on their inverse kinematics (IKs) and motion capture methods, leads to differences in user embodiment [11]. F. Ma studied embodiment based on facial expressions in VR [12]. D. Roth explored the impact of the resemblance of full-body avatars to real individuals on embodiment and social interaction through experiments where two individuals faced each other and performed actions [13]. Elhassan Makled researched differences in embodiment aspects on smart devices with augmented reality (AR) rather than on computers [14]. These studies have provided insights into how differences in appearance and motion of the avatars influence users’ embodiment. Regarding avatar animation techniques, such as the use of inverse kinematics or motion capture, some studies explored the influence of motion artifacts (latency, noise, etc.) in such techniques on the sense of agency, showing, for instance, that they impact the sense of agency but do not break it [15]. Others also explored the impact of such controls on the sense of ownership [13] or on the sense of embodiment [11]. However, no studies have explored, to our knowledge, the influence of control techniques using deep learning on the sense of embodiment.

### 2.2. Neural Networks for a Full-Body Avatar

Many state-of-the-art approaches have achieved full-body motion capture by using six body-worn sensors on the user’s head, limbs, and waist [16,17,18,19,20,21]. Marcard et al. [18] presented an offline method for human motion capture. Huang et al. [19] proposed the first deep learning method, which uses a bidirectional recurrent neural network (RNN) to estimate the human pose in real time. TransPose [20] estimates global translations and body poses from 6 IMUs at 90 fps with state-of-the-art accuracy and was later combined with physics-based motion optimization for joint torque and ground reaction [21]. EM-Pose [22] proposes an approach based on electromagnetic field (EM) sensing.

Research on estimating full-body human motion using even fewer tracking signals often requires a sensor on the waist for flexible lower-body motions. Wouda et al. [23] employed an LSTM-based model to reconstruct the full body from five sensors mounted on a user’s limbs and the waist. LoBSTr [24] used a gated recurrent unit (GRU) model to predict the lower-body motions from four upper-body VR sensors mounted on the head, hands, and waist and to compute the upper body using an IK solver. They state that three sensors make achieving a wide range of motions difficult.

Recently, some studies have reconstructed a full-body avatar using a headset and two hand-held controllers. CoolMoves [25] was the first to use input from only the headset and hand-held controllers. Since they use the template motion repository to synthesize human motion, match similar motions, and interpolate between them, they limit the range of synthesized motions. Dittadi et al. [26] used a variational autoencoder (VAE) framework to compress the head and hands inputs to a latent space, generating a full-body pose by sampling from that latent space. However, the authors assumed that the avatar root is at the origin, and the representation of other joints is relative to the root. Therefore, they implicitly used the pelvis as a fourth input location, which means that they solved the problem from four locations and three rotations of sensors. FLAG [27] proposes an approach based on conditional normalizing flows for sparse inputs to compute the exact pose likelihood, and it outperformed state-of-the-art methods on the AMASS dataset, leading to a deficient error. However, the authors also assumed the avatar root at the origin and had the same problem with a VAE framework. Therefore, a VAE framework and FLAG are only practical in actual data measurement when users stay at a specific location. AvatarPoser [28] employs a Transformer to learn pose features, decouples the global motion from learned pose features, and optimizes the learned features using an IK. Neural3Points [29] combines a data-driven method with physics-based simulation and uses deep reinforcement learning to reconstruct the realistic full-body movement of the user according to three VR trackers. QuestSim [30] incorporates an off-the-shelf physics simulator into the reinforcement learning pipeline to constrain the solution space to physically correct poses. QuestSim produces accurate and plausible simulations by mitigating artifacts such as jitter, foot skating, and unstable contacts. However, these reinforcement learning approaches have limitations regarding the unexpected users’ motions in interactions. Moreover, the delay is usually about 100~160 ms, making it impossible to meet real-time VR requirements.

For VR avatars, neural networks generate plausible full-body motions. Compared to the six body-worn motion capture methods, motion reconstruction from three sensors (headset and two hand-held controllers) sometimes produces lower body artifacts, such as foot-skating.

### 2.3. Backgrounds on Inverse Kinematics for an Avatar

IKs methods involve a procedure that determines the movement of kinematic joints based on their end joints and reconstructs the full-body pose from a headset and controllers. A. Aristidou surveyed the research of the methods for utilizing inverse kinematics to resolve the movement of kinematic chains [31]. The Jacobian method utilizes a matrix of partial derivatives of the entire chain system with respect to the angle parameters. The Jacobian method solves the IKs problem by repeatedly changing the configuration of a complete chain to bring the end effector position and orientation closer, at each step, to a target position and orientation. The cyclic coordinate descent (CCD) method aligns each joint position with the end effector and the target at each step, starting from the end effector and moving inward towards the manipulator base. The CCD method minimizes position and orientation errors by transforming one joint variable at a time. Forward and backward reaching IKs (FABRIKs) works in a forward and backward iterative mode, which minimizes the distance between the target and the end effector each time.

For VR avatars, IKs replicate the upper-body pose of the avatar from a given headset and hand-held controllers. While the upper-body pose is plausible, the lower-body pose is unnatural due to IKs’ limitations.

## 3. Experiments

**Figure 1:**
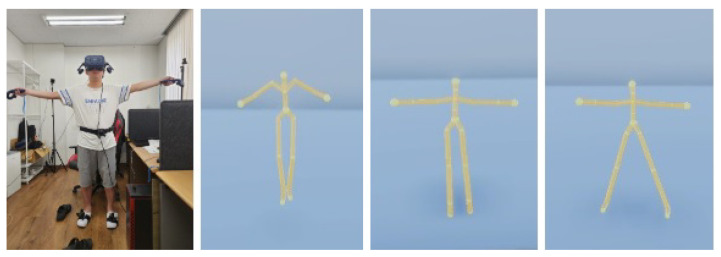
VR user high-, mid-, and low-control avatars from (**left**) to (**right**), respectively.

The experiment aims to investigate the effect of the avatar’s control methods and the assigned tasks on the perceived sense of embodiment (location, agency, and ownership). Downstream of this, we are interested in determining if the higher the control is, the higher the embodiment is in all the kinds of assigned tasks. Participants wore a head-mounted display (HMD), held two hand-held controllers, and were attached to three trackers. We used three kinds of control methods to generate a full-body avatar following the motion of the participants in VR: the low control, where an upper body follows a user’s motion and a lower body floats; the mid control, where full-body motion is generated from the head and two hand-held sensors; and the high control, where full-body motion is generated from the six body-worn sensors, as shown in Figure 1. We conducted two VR experiments: single-user and multi-user experiments. In these two experiments, we investigated the influences of the levels of motion control on the sense of embodiment.

### 3.1. Avatar Control

The users wear the head-mounted devices and hold two controllers in their hands. Let $s_{s,t}^{w}$, a 3 by 1 vector, be the position of *s* sensor at *t* timeframe in a world frame and $O_{s,t}^{w}$, 3 by 3 matrix, be the orientation of *s* sensor at *t* timeframe in a world frame. Given a set of sensor positions and orientations, ${\left\{s_{s,t}^{w},O_{s,t}^{w}\right\}}^{s=1:3}$ in the Cartesian coordinate system, we find the head and wrist joints of the body model, ${\left\{p_{j,t}^{w},R_{j,t}^{w}\right\}}^{j=head,lw,rw}$, where $p_{j,t}^{w}$ and $R_{j,t}^{w}$ are the position and orientation of *j* joint at *t* timeframe in a world frame. We compute the translational and rotational displacements between these sensors and the corresponding joints of our avatar model. Let the sensors’ x,y, and *z* axes point toward the sensors’ right, upward, and forward directions in a virtual world. The position of the head joint is translated backward at about $d_{1}$, which is the approximate distance between the head and the headset. The position and orientation of the head joint is

(1) $$\begin{array}{cc} p_{head,t}^{w} & =s_{1,t}^{w}-d_{1}z_{1,t}\\ R_{head,t}^{w} & =O_{1,t}^{w} \end{array}$$

where $z_{1}$ is the basis vector for the headset’s *z*-axis forward directional vector at *t* timeframe. The positions of the left and right wrists are translated in the right and left directions of the corresponding controllers at about $d_{2}$, which is the approximate distance between the controller and the wrist joint. The position of the left wrist, $p_{lw,t}^{w}$, and the position of the right wrist, $p_{rw,t}^{w}$, at a *t* timeframe are computed from the position of the left controller, $s_{2,t}^{w}$, and that of the right controller, $s_{3,t}^{w}$, as such:

(2) $$\begin{array}{cc} p_{lw,t}^{w} & =s_{2,t}^{w}+d_{2}x_{2,t}\\ p_{rw,t}^{w} & =s_{3,t}^{w}-d_{2}x_{3,t} \end{array}$$

where $x_{2,t}$ and $x_{3,t}$ are the right directional vectors of the left and right controller at the *t* timeframe. The orientations of the wrist joints are computed using the rotational displacements from the corresponding controllers at setup time. The orientation matrix of the left wrist, $R_{lw,t}^{w}$, and the right wrist, $R_{rw,t}^{w}$, is as defined follows:

(3) $$\begin{array}{cc} R_{lw,t}^{w} & =O_{2,t}^{w}{\left(O_{2,1}^{w}\right)}^{T}R_{lw,1}^{w}\\ R_{rw,t}^{w} & =O_{3,t}^{w}{\left(O_{3,1}^{w}\right)}^{T}R_{rw,1}^{w} \end{array}$$

Computed positions and orientations of the head and wrist joints, ${\left\{p_{s,t}^{w},R_{s,t}^{w}\right\}}^{s=head,lw,rw}$ in Equations (1)–(3), represent the head and wrist joints with respect to the world frame in a virtual world. To associate the values to be posturally meaningful, we need to convert data of the articulated joints with respect to the reference frame of a body model. Typical reference frames for human bodies have the pelvis joint as the origin, but VR applications have no information about the pelvis joint. Therefore, we defined the reference frame of a body model using three computed joints.

Our reference frame for an avatar defines an origin, $o_{t}^{a}$, by projecting the head joint on the ground such that

(4) $$o_{t}^{a}=p_{head,t}^{w}-\left(p_{head,t}^{w}\cdot y^{w}\right)y^{w}$$

where $y^{w}$ is the unit normal vector of a ground plane with respect to the world frame. Specifically, the ground plane is a xz plane calibrated at setup time. The basis vectors of our avatar frame align to the body’s right, upward, and forward directions. We fix the upward direction, $y^{a}$, for a stable reference frame by setting a ground plane normal $y^{w}$ as one coordinate direction, which is defined as

(5) $$y^{a}=y^{w}.$$

We find two other orthogonal directions that we call $x_{t}^{a}$ and $z_{t}^{a}$ by approximating the shoulder direction derived from the three joints. We obtain the candidate direction to the avatar’s right shoulder, ${\tilde{x}}^{a}$, as follows:

(6) $${\tilde{x}}_{t}^{a}={\hat{d}}_{rw}-{\hat{d}}_{lw}$$

where ${\hat{d}}_{rw}$ and ${\hat{d}}_{lw}$ are the unit normal vector of the projected displacements on the xy plane of the left and right wrists, $d_{rw}$ and $d_{lw}$, from the origin.

(7) $$\begin{array}{cc} d_{rw} & =p_{rw,t}^{w}-\left(p_{rw,t}^{w}\cdot y^{w}\right)y^{w}-o_{t}^{a}\\ d_{lw} & =p_{lw,t}^{w}-\left(p_{lw,t}^{w}\cdot y^{w}\right)y^{w}-o_{t}^{a} \end{array}$$

The forward direction, $z_{t}^{a}$, must be orthonormal to both ${\tilde{x}}_{t}^{a}$ and $y_{t}^{a}$ and is found by taking the cross-product:

(8) $$z_{t}^{a}={\tilde{x}}_{t}^{a}/∥{\tilde{x}}_{t}^{a}∥\times y_{t}^{a}.$$

The right direction is then

(9) $$x_{t}^{a}=y_{t}^{a}\times z_{t}^{a}$$

Our reference frame for an avatar is robust under VR users’ looking around and bending their arms significantly.

Using the reference frame of an avatar, we enable distinguishing translation and rotation of an avatar instance in the world frame and representing the pose with respect to the avatar frame. The position and orientation of the head and wrist joints in the avatar frame, ${\left\{p_{j,t}^{a},R_{j,t}^{a}\right\}}^{j=head,lw,rw}$, are

(10) $$\begin{array}{cc} p_{j,t}^{a} & =scaleM_{t}\left(p_{j,t}^{w}-o_{t}\right)\\ R_{j,t}^{a} & =M_{t}R_{j,t}^{w} \end{array}$$

Here, scale is a user-specific scale factor, so that the height of the user is scaled to 1.0, and rotation matrix $M_{t}$ has the basis vector of the reference frame as row vectors. The position and orientation of the head and two wrist joints have representations with respect to a reference frame of an avatar, which changes as the user moves around.

#### 3.1.1. Low-Control Avatar

We applied IKs to reconstruct the upper-body pose of the avatar from the headset and two hand-held controllers. Since the headset and two hand-held controllers are the end effector joints in the kinematic chains of the upper body, the IKs method generates the upper-body joints from these end effectors. Using the IKs method, the upper body of the avatar follows the user’s pose and translates and rotates in the virtual world as the user moves in the physical world. The resulting upper-body pose is natural and plausible in a wide variety of end effector inputs. There are numerous possible poses for the lower body. Instead of imagining the lower-body pose, we adopted the avatar’s default standing posture. When the avatar is in motion, the upper body replicates the user’s motion, and the lower body translates and rotates depending on the reference frame of the root joint. We used the Final IKs asset [32] to implement the upper-body avatar from the headset and two hand-held controllers.

#### 3.1.2. Mid-Control Avatar

Given the position and orientation of three VR sensors, we achieve the positions and rotations of the articulated joints for a full body by learning a mapping function $f:R^{36}\to R^{264}$ through the following equation:

(11) $$\left(p_{0,t},R_{0,t},{\left\{R_{j,t}^{l}\right\}}_{t=1:t_{n}}^{j=1:21}\right)=f\left({\left\{p_{j,t},R_{j,t}\right\}}_{t=1:t_{n}}^{j=head,lw,rw}\right)$$

where $p_{0,t}$ and $R_{0,t}$ are the global position and orientation of the pelvis joint with respect to the avatar reference frame, $R_{j,t}^{l}$ define the local position and orientation of the 21 articulated joints, and $t_{n}$ matches the number of observed VR frames considered from the past. Our SMPL body model excludes the last two joints and uses 22 joints, whose indexing ranges from 0 to 21.

The input is the position $p_{j}^{a}$, velocity $v_{j}^{a}$, rotation matrix $R_{j}^{a}$, and angular rotation matrix $W_{j}^{a}$ from the head and two wrist joints in an avatar frame. We use a rotation matrix and angular rotation matrix, discard the first row, and end up with a continuous 6D representation, $\theta_{j}^{a}$, a 6 by 1 vector, and $\omega_{j}^{a}$, also a 6 by 1 vector. The final input representation is a concatenated vector of the position, velocity, rotation, and angular rotation from all given sparse inputs, $X\in R^{54}$, which is defined as follows:

(12) $$X={\left\{{\left(p_{j}^{a}\right)}^{T},{\left(\theta_{j}^{a}\right)}^{T},{\left(v_{j}^{a}\right)}^{T},{\left(\omega_{j}^{a}\right)}^{T}\right\}}^{j=head,lw,rw}$$

Note that these inputs in *X* associate the global values in the avatar frame.

The output is the global position and orientation of the pelvis joint, j=0, and the local rotation of the articulated joints, j=1,⋯,21, with respect to their parent joints. Our output representation is a concatenated vector from the pelvis and the other 21 joints, $Y\in R^{135}$, as follows:

(13) $$Y=\left[{\left(p_{0}^{a}\right)}^{T},{\left(\theta_{0}^{a}\right)}^{T},{\left(\theta_{1}^{l}\right)}^{T},\cdots ,{\left(\theta_{21}^{l}\right)}^{T}\right]$$

We applied the Transformer Encoder to process the time sequences of the pose data and extract features, relying entirely on an attention mechanism to draw global dependencies between input and output, similar to AvatarPoser [28]. Specifically, given the input signals, we applied a feedforward network to embed features from 54 to 256 dimensions linearly. Next, our Transformer Encoder extracted pose features from previous time sequences of the headset and hands. Two-layer feedforward networks were used to decode features to 256 and 135, respectively. We set the number of heads at 8 and the number of self-attention layers to 3.

The final loss function comprises an L1 loss for the pelvis position, an L1 local rotational loss, and an L1 forward kinematics loss, which is denoted as:

(14) $$L=\lambda_{1}L_{pelvis}+\lambda_{2}L_{rot}+\lambda_{3}L_{fk}$$

where the hyperparameters are $\lambda_{1}=0.05$, $\lambda_{2}=1.0$, and $\lambda_{3}=1.0$. The pelvis position loss (Equation (15)) measures the L1 norm, where the ${\hat{p}}_{0}^{a}$ and $p_{0}^{a}$ are the estimated value and the ground truth of pelvis position. This loss term encourages accurate estimation of the pelvis position:

(15) $$L_{pelvis}=\left|\right|{\hat{p}}_{0}^{a}-p_{0}^{a}{\left|\right|}_{1}$$

The rotation loss (Equation (16)) contributes to the overall loss function by quantifying the L1 norm between the estimated value ${\hat{\theta }}_{s}^{j}$ and the ground truth 6D rotation matrix $\theta_{j}^{a}$ for the *j*th joint. This term promotes the alignment of estimated joint orientations with the ground truth orientations, leading to an accurate estimated pose.

(16) $$L_{rot}=\left|\right|{\hat{\theta }}_{j}^{l}-\theta_{j}^{l}{\left|\right|}_{1}$$

The FK loss (Equation (17)) is an L1 positional loss, where ${\hat{p}}_{j}^{a}$ and $p_{j}^{a}$ define the ground truth position and the estimated joint position for the *j*th joint:

(17) $$L_{fk}=\left|\right|{\hat{p}}_{j}^{a}-p_{j}^{a}{\left|\right|}_{1}$$

#### 3.1.3. High Control Avatar

Motion capture methods require a minimum of six sensors to capture a full-body pose [21,33]. Six sensors are attached to the end-effector joints of an articulated human body model. The human body is an articulated model with the pelvis joint as its root joint, and the upper and lower bodies are two children of the pelvis joint. According to the kinematic chain, the end effector joints of the upper-body part are the head and two hand joints, and those of the lower-body part are the two foot joints. Therefore, we exploited the headset and two hand-held trackers of the VR devices for the end effector joints of the upper-body part and attached additional trackers to the belly button of the participant and the top of the left and right feet for the end effector joints of the lower-body part. Six sensors provide the position and rotation of the root joint and all end effector joints in the kinematic chain. We defined the global location and orientation of the avatar from the pelvis tracker and reconstructed the full-body pose from the end effectors: headset, two hand-held controllers, and two foot trackers.

### 3.2. Experimental Design

This experiment aimed to study the contribution of control toward the sense of embodiment. In other words, users would significantly enhance their sense of embodiment toward an avatar of accurate control. To this end, we formulated the following hypotheses:

- H1: The low-, mid-, and high-control avatars demonstrate a similar sense of embodiment.
- H2: The mid- and high-control avatars demonstrate a similar sense of embodiment.
- H3: The interaction task using a controlled avatar with another virtual body brings a similar sense of embodiment to a first-person task.

We used a within-subject design to test our hypotheses. We split the study into two tasks: a single-player experiment in which participants saw their avatars in the mirror and a multi-player experiment in which participants saw others’ avatars.

#### 3.2.1. Single-User Experiment

**Figure 2:**
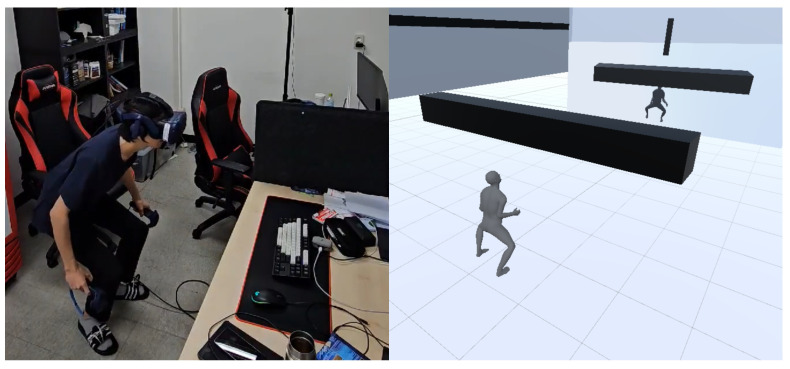
Obstacle task. A crouching participant in a physical world (**left**) and the avatar from the third-person perspective in VR (**right**).

In the single-user experiment, the obstacle game, participants must use their bodies to avoid the obstacles. Participants can see their avatars reflected in the front mirror and receive visual feedback on their avatars as shown in Figure 2. Black rectangular obstacles originating from a far distance are moving toward the participants. Black rectangular obstacles have a vertical form and a horizontal form. Participants must crouch to avoid vertical obstacles and move left or right to avoid horizontal obstacles. The task is to avoid ten obstacles for 30 s. Figure 2 is the participant on the left image, and the virtual avatar and obstacles on the right image have been captured from the third-person perspective in VR.

#### 3.2.2. Multi-User Experiment

**Figure 3:**
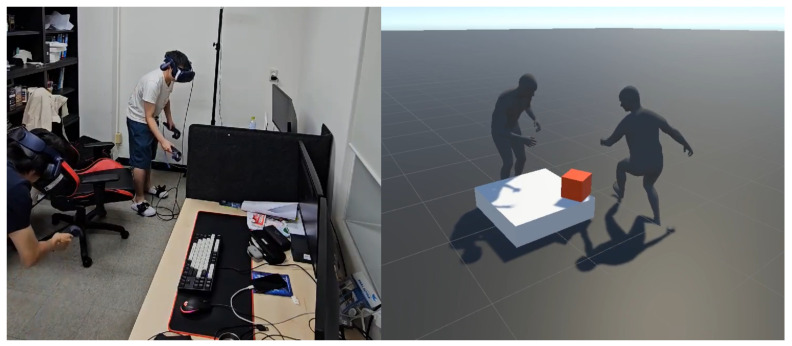
Catch ball task. Two participants pass the cube in a physical world (**left**) and as corresponding avatars from the third-person perspective in VR (**right**).

In the multi-user experiment, the catch-ball, two participants need to use their arms and move around to throw and receive the objects to each other. Figure 3 shows how they throw and receive a cube-shaped object to each other. Figure 3 shows one participant on the left image, and the catch-ball game on the right image has been captured from the third-person perspective in VR. The task is to throw and catch the ball six times for 30 s.

### 3.3. Experiment Protocol

We initially recruited 20 participants for the user study. All participants were aged between 22 and 29. A total of 40% of the participants were female, all were right-handed, and 55% had had VR experiences.

We began with a simple demographic questionnaire. Then, we conducted a single-user experiment. Participants were instructed to perceive the environment for 10 s and then avoid obstacles for 30 s. The obstacle avoidance task was repeated three times for the low, medium, and high avatars. The order of avatars was randomly selected. Next, the multi-player catch-ball task was conducted. Participants were asked to perceive the surrounding environment for 10 s and perform throwing three times. The order of avatars was randomly selected. We tracked each participant’s performance during both tasks by recording the hits of obstacles in the obstacle task and dropped balls in the catch-ball task.

After each experiment, we asked participants to answer a questionnaire with eight questions on a five-point Likert scale from 1 (totally disagree) to 5 (totally agree). Questions Q1 to Q8 are the embodiment questions designed to study participants’ feelings of embodiment in each condition. Questions Q1 and Q2 contain questions related to the sense of self-location; questions Q3, Q4, and Q5 contain questions related to the sense of agency; and questions Q6, Q7, and Q8 contain questions related to the sense of ownership. After completing all the conditions of one task, we asked participants to rate their preferences for all the conditions on a five-point Likert scale concerning embodiment (Q9), preference for the fast game (Q10), and overall experience (Q11).

## 4. Results

**Figure 4:**
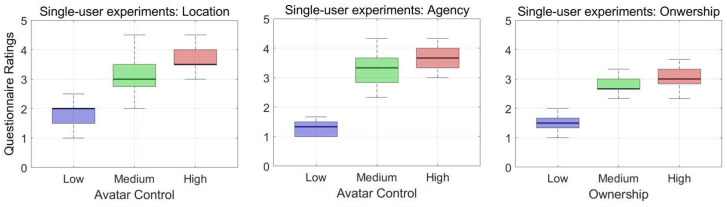
A boxplot of embodiment ratings from the single-user experiment: the perceived sense of location (**left**), agency (**center**), and ownership (**right**) for each avatar control obtained through Q1–Q2, Q3–Q5, and Q6–Q8, respectively (from 0 to +5).

**Figure 5:**
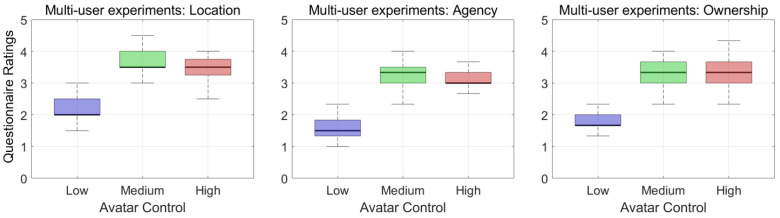
A boxplot of embodiment ratings from the multi-user experiment: the perceived sense of location (**left**), agency (**center**), and ownership (**right**) for each avatar control obtained through Q1–Q2, Q3–Q5, and Q6–Q8, respectively (from 0 to +5).

We used a total of 20 datasets for the statistical analysis. Since the normality assumption (Shapiro–Wilk’s Normality test) was violated, a Friedman test was conducted, followed by a post hoc Wilcoxon signed-rank test. We report the significant differences here (p< 0.05). The questionnaire data for embodiment are presented in Figure 4 and Figure 5. Questionnaire ratings were averaged for the sense of location (Q1 and Q2), agency (Q3, Q4, and Q5), and ownership (Q6, Q7, and Q8) for both the single-user and multi-user experiments.

### 4.1. Control Comparison

The questionnaire results show that the median scores of the low-control avatar were lower than those of the mid- and high-control avatars in both experiments. The median scores of the mid-control avatar were lower than those of the high-control avatar in the single-user experiment and higher than those of the high-control avatar in the multi-user experiment.

We conducted Friedman’s two-way analysis of variance by ranks on the embodiment scores to compare the three kinds of avatar control methods, and the results are shown in Table 1. In the single-user experiment, the average ranks of the low-, mid-, and high-control avatars came out to 1.0, 2.08, and 2.93, respectively, and these results reject hypothesis H1 (*p* < 0.001), showing significant differences. In the multi-user experiment, the average ranks of the low-, mid-, and high-control avatars came out to 1.0, 2.5, and 2.5, respectively, and these results reject hypothesis H1 (*p* < 0.001).

**Figure 6:**
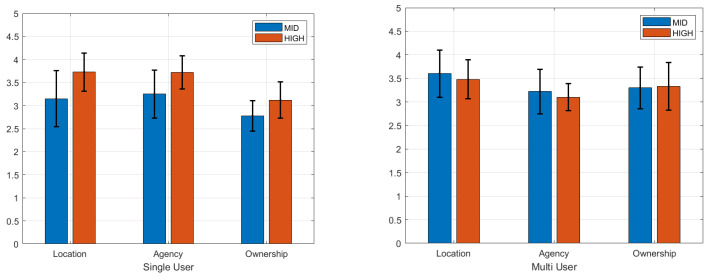
The mean scores for the sense of location, agency, and ownership regarding the mid- and high-control avatars: mean scores in the single-user experiment (**left**) and mean scores in the multi-user experiment (**right**).

We conducted pairwise signed-ranks tests as a post hoc analysis of the Friedman test to pinpoint which pairwise groups have a significant difference. The pairwise comparison in Table 2 shows the standardized test statics (stats), Bonferroni-corrected significant probability (p), and significant differences (sig) for the pairwise groups in the single-user and multi-user experiments. While other pairwise groups yielded significant differences, the comparison of the mid- and high-control avatars yielded no significant difference in the multi-user experiment, accepting hypothesis H2, which was different from the single-user experiment. Since the pairwise comparisons of the mid- and high-control avatars yielded different results in the single-user and multi-user experiments, we investigated the three elements of the sense of embodiment: self-location, agency, and ownership. Figure 6 displays the mid- and high-control avatars in the single-user experiment on the left and those in the multi-user experiment on the right. The pairwise comparison shows significant differences for the all the location, agency, and ownership scores in the single-user experiment. It shows no significant differences in the multi-user experiment’s location, agency, and ownership scores.

### 4.2. Task Comparison

**Figure 7:**
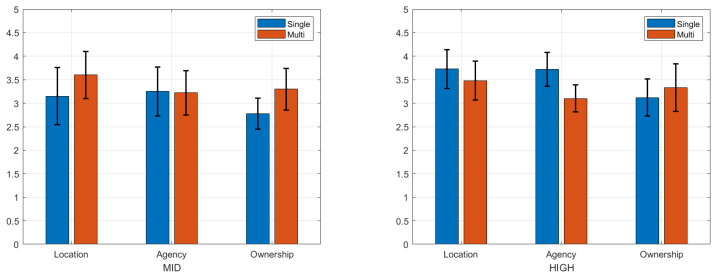
Comparison of the single- and multi-user tasks of the mid- and high-control avatars.

We performed the Mann–Whitney U test on embodiment scores between tasks: the single-user and multi-user experiments. Table 3 shows our statistical analysis comparing the single and multi-user tasks regarding the sense of embodiment and the three components of the sense of embodiment: a sense of self-location, a sense of agency, and a sense of ownership. All of the kinds of control yielded significant differences in the sense of embodiment between the single- and multi-user tasks, rejecting hypothesis H3. Looking closely at the components, the analysis of the low-, mid-, and high-control avatars portrays a different appearance. The low-control avatar shows a significant difference in agency and ownership, the mid-control avatar does so in terms of location and ownership, and the high-control avatar does so in terms of agency. Figure 7 shows the mean and standard deviations of the mid-control avatar on the left and the high-control avatar on the right. The mid-control avatar on the left demonstrated an enhanced sense of ownership in the multi-user task, while the high-control avatar on the right registered a decreased sense of agency.

### 4.3. Post-Questionnaire Results

**Figure 8:**
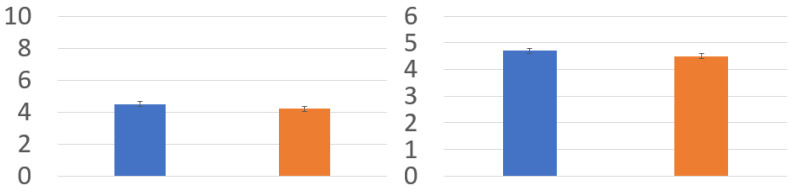
The average number of successful avoidances of obstacles during the obstacle task (**left**) and the average number of successful passes during the catch-ball task (**right**). Blue and orange colors represent the mid- and high-control avatars, respectively.

The left graph in Figure 8 shows the average number of obstacles that participants avoided in the obstacle task, and the right graph in Figure 8 shows the average number of balls that participants caught in the catch ball task. On average, participants avoided 4.5 out of 10 obstacles using the medium-control avatar and 4.1 using the high-control avatar. In the catch-ball task, participants caught 4.7 out of 6 balls using the medium-control avatar and 4.4 using the high-control avatar.

**Figure 9:**
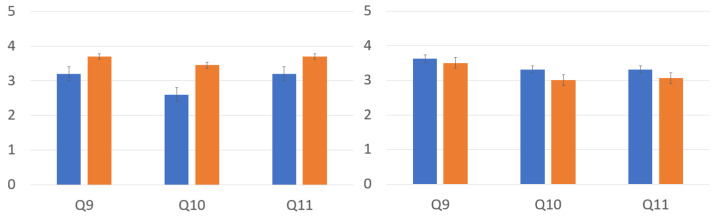
Preference questions: The mean scores of questions Q9, Q10, and Q11 for each avatar for obstacle task (**left**) and the mean scores of questions Q9, Q10, and Q11 for each avatar for catch ball task (**right**). Blue and orange are the mid- and high-control avatars, respectively.

The final preference questions were asked after the participant finished all the experiments. The results in Figure 9 are the preference question results in the obstacle task (left) and the results in the catch ball task (right). In the obstacle task, all participants rated the high-control avatar higher; in the catch-ball task, all participants rated the mid-control avatar higher. The pairwise test shows that preferences between the mid- and high-control groups show no noticeable differences in both tasks.

## 5. Discussion

### 5.1. One’s Own Control and Others’ Control

According to our results, improving the control of the avatar affects the sense of embodiment. All task participants primarily reflect this, as they scored significantly lower for the low-control avatar in all aspects of embodiment than the other mid- and high-control avatars. This is also visible in the preference questions, where no participants chose the low-control avatar. While an unfavorable preference for the low-control avatar was reflected in all tasks, the comparisons of the mid-control and high-control avatars report different results in the single-user and multi-user experiments. In the single-user experiment, improving the control of the avatar from mid to high enhanced the sense of embodiment. In the multi-user experiment, however, the mid-control and high-control avatars reported no significant differences in embodiment and preference. This reveals an uncanny valley-like effect of the degree of avatar control in the multi-user experiment, which is unlike in the single-user experiment. The difference in the effects of improving the control between the single-user and multi-user experiments might come from the virtual bodies that the participants control and see as being the same or different. While the participants see the virtual body reflected in the mirror that they control in the single-user experiment, they see the virtual body that another participant controls in the multi-user experiment. Although the high-control avatar follows the participant’s motion more precisely than the mid-control avatar, it makes no difference in embodiment when one sees the virtual body that others control. Overall, these results underline that participants expect precise control of their own virtual bodies and accept the plausible control of others’ virtual bodies. The intriguing result is that the mid-control avatar using neural networks could be an alternative to the high-control avatar using the six wearable sensors when visualizing other participants’ virtual bodies. While the effectiveness of improving control of one’s own and others’ virtual bodies has partially been explored, further research would be needed to better understand this result.

### 5.2. Influence of the Interaction Task

We describe our other results testifying to the influence of the task on the sense of embodiment. Comparison between the single-user experiment—the obstacle-avoidance task—and the multi-user experiment—the catch-ball task—hows that they have different distributions among all the kinds of avatar control. According to our results in Table 3, the catch-ball task increased the sense of embodiment of the low-control avatar with the largest significant probability (p<0.001), which was followed by the mid-control avatar (p=0.002). The main difference in the catch-ball task is seeing and interacting with others’ virtual bodies, and therefore, seeing and interacting with others’ virtual bodies was found to enhance the sense of embodiment. It can be seen above that the catch-ball task yielded an enhanced sense of agency and ownership of the low-control avatar compared to the first-person obstacle-avoidance task. It is also noticeable that the catch-ball task enhanced the sense of location and ownership of the mid-control avatar. On the other hand, the catch-ball task, however, decreased the sense of embodiment of the high-control avatar significantly (p=0.012). This decreased sense of embodiment is because of the significantly different sense of agency. It is a rather paradoxical result that seeing and interacting with others’ virtual bodies decreases the sense of embodiment of the high-control avatar. Overall, interacting with others’ virtual bodies influences the sense of embodiment, and the influence is either positive or negative, depending on the degree of avatar control. This is an intriguing result, since social interaction in VR is known to have a strong positive impact on the sense of embodiment.

The sense of ownership appeared to be the most enhanced sense among the component of the sense of embodiment when performing the catch-ball task compared to when performing the obstacle-avoidance task. This is primarily reflected in the low- and mid-control avatar experiments in terms of task comparison, as their significant probabilities came out to 0.004 and 0.001, while the high-control avatar demonstrated a similar sense of ownership. The sense of location seemed to be the stable factor, showing their significant probabilities ranging around the borderline between the single- and multi-user tasks in all degrees of control. The sense of agency appeared to be a controversial component of the sense of embodiment between the obstacle-avoidance task and the interacting catch-ball task. This is notable in the high-control avatar, where the catch-ball interaction decreased the sense of agency with *p*-value (<0.001). The social interaction decreased the sense of agency with the high-control avatar, which was not visible with the low- and mid-control avatars. We may then wonder why the sense of agency was decreased during that task. While our results do not allow us to answer this question, it is essential to consider the potential influences of the characteristics of the multi-user experiment, such as interaction with another user by throwing and catching the balls. For instance, while participants were precisely instructed to catch a ball thrown by another participant, their failure to catch a ball could still have been interpreted as a failure of agency by the participants. Participants experience revulsion when encountering failure when it seems possible. Therefore, a possible explanation could be that participants associated the increased degree of avatar control with an increased chance of catching a ball, feeling unease, and yielding revulsion by accepting failure more seriously when they experienced missing a ball.

While it is difficult to observe a specific pattern of influence depending on the task, such as standalone and interacting with another, the results demonstrate that the scores of embodiment and especially agency were not the same in both tasks. This outcome could raise questions about whether task performance impacts the sense of agency. For this reason, it would be very interesting to further investigate whether the type of task influences the sense of agency independently.

### 5.3. Limitations

The inter-relations between the degree of control and interaction tasks influencing the sense of embodiment are complex. While we tried to address the question of user embodiment regarding the degree of control and assigned tasks in this paper, we believe future research would provide more insights on the subject.

In our paper, the experimental design and technical limitations constrained the accuracy of avatar control. In some cases, we may wonder how the limitations in implementation and hardware specifications impacted user embodiment. For instance, the wearing of additional sensors on the body in the high-control avatar may partially explain why participants tended to score similarly in the mid-control avatar. Also, the latency in the multi-user experiment due to network communication may have degraded the motion accuracy, partially explaining why participants tended to score the mid- and high-control avatars similarly.

Furthermore, we may consider the potential influence of task characteristics. In the single-user experiment, the obstacle-avoidance task, the simplicity of the obstacle shapes and cubes might have resulted in a lack of perceived threat. The results would differ if objects other than the obstacles were employed to induce a sense of threat. In the multi-user experiment, the difficulty of catching and throwing balls might have negatively influenced the sense of agency. Future research on easy and difficult tasks would, we believe, provide more insights into interaction tasks and the sense of embodiment. Additionally, when the experiment’s duration is longer than 30 s, this might also yield distinct results.

## 6. Conclusions

In this paper, we studied the effects of avatar control on embodiment through low-, mid-, and high-control avatars. The low-control avatar consisted of the animating upper body and the floating lower body. The mid-control avatar reconstructed an entire body from the head and two hand-held controllers. The high-control avatar generated an entire body from the head, two hand-held controllers, the pelvis, and two on-foot sensors. We used three kinds of control for the avatar and conducted single-user and multi-user experiments on embodiment. Firstly, integrating full-body movements into avatars significantly influences the sense of embodiment in VR. The low-control avatar consistently received lower scores in questions measuring overall embodiment, indicating that the existence of full-body movements enhances embodiment in VR. Secondly, the mid-control and high-control avatars demonstrated no significant difference in the sense of embodiment regardless of the accuracy of the control method. This shows an uncanny valley effect: The high-control avatar decreased the sense of agency in the multi-user experiment, which required precise control of the avatar. Further research and exploration under different task requirements are necessary to uncover avatar control’s effects on embodiment.

## Figures and Tables

**Table 1:** Friedman’s two-way analysis of variance by ranks of single-user and multi-user experiments. Here, N, DF, *p*, and sig represent sample size, degree of freedom, *p*-value, and significant differences.

Test	N	Chi-Square	DF	*p*	sig
single-user	20	37.696	2	<0.001	yes
multi-user	20	31.579	2	<0.001	yes

**Table 2:** Friedman test post-hoc analysis of single-user and multi-user experiments. Here, stats, *p*, and sig are standardized test statistics, Bonferroni-corrected *p*-value, and significant differences.

Single-User					Multi-User				
**Group1**	**Group2**	**Stats**	***p***	**sig**	**Group1**	**Group2**	**Stats**	***p***	**sig**
Low	Mid	−3.399	0.002	yes	Low	Mid	−4.743	0.000	yes
Low	High	−6.087	0.000	yes	Low	High	−4.743	0.000	yes
Mid	High	−2.688	0.022	yes	Mid	High	0.000	1.000	no

**Table 3:** Mann–Whitney test analysis between the single-user and multi-user tasks, where *p* is significant probability; *p*-value measurements are of the low-, mid-, and high-control avatars regarding the sense of embodiment and three components of the sense of embodiment; and sig a is significant difference between the single-user and multi-user tasks.

	Embodiment	Location	Agency	Ownership
	*p* (sig)	*p* (sig)	*p* (sig)	*p* (sig)
low	<0.001 (yes)	0.056 (no)	0.043 (yes)	0.004 (yes)
mid	0.002 (yes)	0.017 (yes)	0.904 (no)	<0.001 (yes)
high	0.012 (yes)	0.114 (no)	<0.001 (yes)	0.192 (no)

## Data Availability

The data that support the findings of this study are available from the corresponding author, [K.P.], upon reasonable request.

## Abbreviations

The following notations and abbreviations are used in this manuscript:

IKs	Inverse Kinematics
VR	Virtual Reality
AR	Augmented Reality
CCD	Cyclic coordinate descent
